# Physical therapy for patients with low back pain in Germany: a survey of current practice

**DOI:** 10.1186/s12891-021-04422-2

**Published:** 2021-06-19

**Authors:** Carolin Bahns, Lisa Happe, Christian Thiel, Christian Kopkow

**Affiliations:** 1 Department of Therapy Science I, Brandenburg Technical University Cottbus – Senftenberg, Universitätsplatz 1, 01968 Senftenberg, Germany; 2 Department of Applied Health Sciences, Division of Physiotherapy, University of Applied Sciences, Bochum, Germany; 3 Department for Health Services Research, Assistance Systems and Medical Devices, Carl von Ossietzky University Oldenburg, Oldenburg, Germany; 4 Faculty of Sports Science, Training and Exercise Science, Ruhr-University Bochum, Bochum, Germany

**Keywords:** Low back pain, Physical therapy, Guideline, Guideline adherence

## Abstract

**Background:**

Low back pain (LBP) is one of the most common musculoskeletal disorders worldwide. The National Disease Management Guideline (NVL) “Non-specific LBP” is a practical decision-making aid for both physicians and non-medical professionals in Germany to improve quality of health care. Although LBP is the most frequent diagnosis resulting in physical therapy treatment, information on the current management of patients with LBP and guideline adherence is limited. The objective of this study was to evaluate the current physical therapy management of patients with LBP in Germany, and to explore guideline adherence to the NVL “Non-specific LBP” recommendations.

**Methods:**

An online survey among physical therapists working in Germany was conducted based on the recommendations given in the NVL “Non-specific LBP”. Participants were recruited using a snowball sampling approach and invited to complete the questionnaire. Guideline adherence was evaluated by comparing the survey findings with the recommendations of the NVL.

**Results:**

In total, 1361 physical therapists (41 years of age (SD 11); 839 female, 514 male; 16 years of clinical experience (SD 10)) completed the survey.

Fifty percent (*n* = 675) of our respondents adhered to the recommendations of the NVL on the therapeutic diagnostic process, and 72% (*n* = 973) to the recommendations concerning treatment. The guideline adherence across the entire management process (i.e., both diagnosis and treatment) of LBP was 38% (*n* = 513). German physical therapists predominantly provide active interventions, e.g., exercise therapy, but also use interventions with low or conflicting evidence, respectively. Massage and Kinesio Taping were frequently used despite being not recommended. Less than one third of study participants (*n* = 400, 29%) answered to know the NVL or had dealt with its recommendations.

**Conclusions:**

In the management of LBP, overall guideline adherence among German physical therapists was 38% with higher adherence in the treatment section than in the physical therapeutic diagnostic process. Widespread employment of implementation strategies and removal of existing barriers against the application of evidence-based guidelines could facilitate the transfer of scientific evidence into clinical practice and contribute to optimize the quality of health care.

**Trial registration:**

German Clinical Trials Register (DRKS00012607). Registered 04 October 2017.

**Supplementary Information:**

The online version contains supplementary material available at 10.1186/s12891-021-04422-2.

## Background

Low back pain (LBP) is defined as pain or discomfort in the lumbosacral region, localised below the last rib and above the gluteal crease, with or without referred leg pain [1, 2]. While LBP can result from known or unknown abnormalities or diseases [3], in more than 85 % of cases LBP is considered non-specific [4]. While most episodes are short-lasting and without, or with little lasting consequences, recurrent episodes are common and LBP is increasingly being understood as a long-lasting condition with varying trajectories [3]. As one of the most common musculoskeletal disorders in modern society with a global point prevalence of 9.4 % [5], LBP is a leading cause for disability and work absence [5–7], and causes considerable burden on individuals, their families, the economy, and health care systems [8].

A biopsychosocial approach is recommended for the assessment and management of non-specific LBP, consisting of self-management, physical and psychological interventions, as well as some forms of complementary medicine [9]. Physical therapists play a key role in the management of LBP and interventions offered by physical therapists such as exercise therapy are considered a first-line treatment for chronic LBP [9].

In March 2017, the second version of The National Disease Management Guideline “Non-specific LBP” (in German: Nationale VersorgungsLeitlinie (NVL)) was published based on available evidence and clinical experience [1]. The guideline is intended to be a practical decision-making aid for both physicians and non-medical professionals, e.g. physical therapists, to improve the quality of health care [1]. In contrast to other countries, physical therapy in Germany is still considered an assistant health profession and therapists are only allowed to provide physical therapy services with a physician’s prescription, which is based on the German catalogue of therapies [10].

In a systematic review, Zadro et al. [11] determined guideline adherence of physical therapists when managing musculoskeletal conditions. Based on surveys completed by physical therapists, the median percentage of participants who chose recommended treatments for LBP was 35 %, 44 % for treatments not recommended, whereas treatments with an open recommendation were provided by 72 % of physical therapists. According to Hanney et al. [12], early evidence-based treatment can advance the recovery of patients with LBP and reduce health care utilization and costs. However, physical therapists rarely seem to follow evidence-based guidelines when managing musculoskeletal conditions. Perceived barriers to using guidelines in clinical practice include, for example, lack of time, poor availability, and limited access to guidelines [13].

The systematic review by Zadro et al. [11] does not include a single study from Germany and overall there is little research on the current physical therapy management for musculoskeletal disorders in Germany [14, 15]. The results of this review cannot simply be applied to the German health care system, since unlike in Germany, physical therapists in other countries, e.g. the Netherlands, Great Britain or the USA, are more independent in their decision about physical therapy treatments as they are not bound to a physician’s prescription. An investigation of the physical therapy management in Germany is necessary to identify possible overuse, underuse and misuse of physical therapy, and to explore barriers to using guidelines specific to its health care system.

The objective of this study was to evaluate the current physical therapy management of patients with LBP in Germany, and to explore guideline adherence to the NVL “Non-specific LBP” recommendations. As an academic level of physical therapy education is not mandatory in Germany, and the German physical therapy education does not meet the requirements of direct access [16], we hypothesized guideline adherence in German physical therapists to be low.

## Methods

The study was a priori registered with the German Clinical Trials Register (DRKS-ID: DRKS00012607), which is linked to the International Clinical Trials Registry Platform from the World Health Organization [17]. Ethical approval was obtained from the ethics committee of the German association of physical therapists (Deutscher Verband für Physiotherapie e. V., Ethics Committee No.: 2017-08).

### Study design

For the reporting of this study, the Checklist for Reporting Results of Internet E-Surveys (CHERRIES) [18] was used.

This cross-sectional study was conducted as a nationwide open online survey among physical therapists. Data were collected between October and December 2017. Study participants received written information about purpose, extent, and data storage of the study. By initiating the survey, participants gave informed consent for data analysis and publication. Completion of the survey via a survey link was voluntary, with no incentives offered. Participation was anonymous, and participants had the option of declining to answer specific questions or to leave the questionnaire blank.

### Questionnaire

A self-administered questionnaire was developed based on the NVL “Non-specific LBP” chapters on diagnostics and non-pharmacological therapy (see Additional file 1 on the eAddenda for the complete online survey in German language and Additional file 2 for a translated English language version of the questionnaire). Although in international guidelines a core recommendation is to advice patients to stay active and educate patients to support self-management [19, 20], we did not include this chapter of the NVL in our study. Advice and education should primarily be provided by physicians in Germany since not addressed by the German catalogue of therapies [10], acknowledging that physical therapists might give advice and educate patients within physical therapy sessions in an informal way. This questionnaire served to collect information on (1) participants’ demographics, (2) the physical therapeutic diagnostic process of LBP, (3) the treatment of LBP and (4) the application of clinical practice guidelines (CPG) and perceived barriers. We used a mix of multiple-choice questions and yes/no-questions. For all items, there was a non-response option. To assess the current clinical practice, study participants were asked to rate different treatment modalities for acute and chronic LBP on a 4-point Likert-type scale (where 1 = never, 2 = sometimes, 3 = often, 4 = always). Items were listed alphabetically to avoid any influence of display order. Questions regarding participant’s awareness of the NVL and the application of CPGs in general were developed by the authors. Respondents who denied using guideline recommendations in clinical practice were asked for their perceived barriers based on common barriers described in the literature [21].

For the online questionnaire, SoSci Survey was used, a free of charge online tool for research projects (www.soscisurvey.de). The survey was accessible online without restrictions (password or registration) via an internet link to the SoSci Survey platform. Study participants were estimated to be able to complete the questionnaire within 10–15 min. The sequence of items and thus, the number of pages and items per page, were individually adjusted using filter questions. The maximum number of pages was 27. In case of missing answers, participants were reminded to complete all questions before submission. To ensure an accurate data collection process and to avoid bias, there was no possibility to go back to the previous pages once participants had proceeded to the next page. Technical conditions did not allow determining unique visitors of the survey.

To improve quality and understanding of the questionnaire, a pre-test was conducted with 10 physical therapists. Pre-test participants were asked to point out any difficulties in understanding, semantics, conception or layout. All results obtained in this process were discussed within the study team, resulting in minor adjustments.

### Participants

We recruited physical therapists who had been practicing in Germany, were at least 18 years of age, and were able to read and speak the German language.

The recruitment of participants was initiated through announcements and calls on different physical therapeutic networks, articles in newsletters, social media and relevant internet platforms and personal contacts of the study team. Persons contacted through the different strategies were encouraged to further distribute the participation invitation. Thus, a “snowball sampling” approach has been used. No power calculation was performed due to the exploratory nature of this cross-sectional survey. However, we had targeted a total sample size of 1000 participants to allow regression analyses with subgroups of sufficient sample size. We had not defined a maximum number of participants for the survey.

### Data analysis

Only complete questionnaires were analysed. Data were qualitatively checked for plausibility by two authors and discussed in case of differing assessments.

Statistical analysis was performed using the software R Version 3.3.2 (The R Project for Statistical Computing, Vienna, Austria).

#### Current clinical practice and guideline adherence

Participants’ characteristics and current clinical practice were analysed using descriptive statistics such as frequency distribution and percentages.

Guideline adherence was defined as the accordance between guideline recommendations and the therapists’ treatment or diagnostic choices. Guideline adherence was determined using a point-based system. For both treatment and diagnosis, the benchmark for good adherence was set at ≥ 80 % [22] of the maximally achievable points.

To determine guideline adherence in the physical therapeutic diagnostic process, 23 items were evaluated. The NVL is an interprofessional guideline and contains information for physicians and other health care professionals. Thus, in order to be considered as criteria for guideline adherence in relation to the physical therapeutic diagnostic process, aspects listed in the NVL also needed to be listed in the CPG for LBP published by the Royal Dutch Society for Physical Therapy (KNGF) [20]. Further aspects of the NVL, which were not mentioned in the KNGF guidelines were discussed by the study team and supplemented if they were deemed to be relevant in the German physical therapeutic context.

The categorical data for the questions regarding treatment options were dichotomised. If treatment modalities were recommended in the NVL (↑↑ = strong recommendation for a treatment or ↑ = recommendation for a treatment), the answers ‘always’ and ‘often’ were awarded one point. In case of negative recommendations (↓↓ = strong recommendation against a treatment or ↓ = recommendation against a treatment), the answers ‘never’ and ‘sometimes’ were awarded one point. Treatments with an open recommendation (↔) were not considered in the scoring system. In the treatment section, the highest achievable point score was 32.

Combining guideline adherence of the physical therapeutic diagnostic process and the treatment section, a third dependent variable was defined. Participant’s total guideline adherence was fulfilled if at least 80 % of the maximum number of points in each section had been achieved.

#### Possible determinants in guideline adherence

Associations between participants’ characteristics and their guideline adherence were assessed using exploratory univariate logistic regression and reported as OR and 95 % CI. Guideline adherence was dichotomized in adherence and non-adherence. Potential determinants (sex, highest professional degree, years of professional experience, work setting) were selected based on the literature [13, 22, 23]. The factor `highest professional degree´ is particularly relevant, since in Germany the physical therapy education takes place predominantly at vocational schools (so called “Berufsfachschulen”) and only a few physical therapists (3 %) graduate from higher educational institutions [24]. To consider the specific characteristics and structure of the German health care system, further determinants (size of the city/municipality of employment, interprofessional collaboration) were examined for associations with guideline adherence. Questionnaires with missing values within the analysed variables were excluded from the regression. The level of statistical significance was set at *p*-value < 0.05.

## Results

In total, 1383 physical therapists completed the survey. Twenty-two questionnaires were excluded due to following reasons: no contact with patients with LBP (*n* = 17), working in a country other than Germany (*n* = 2), currently unemployed (*n* = 1), no physical therapist (*n* = 1), no professional degree (*n* = 1). Survey data from 1361 participants were included in the analysis. No data were collected on the view rate, participation rate and completion rate.

### Characteristics of the study population

From the 1361 physical therapists who had completed the questionnaire, 839 (62 %) were women and 514 (38 %) men. The mean age was 41 years (SD 11), the mean clinical experience 16 years (SD 10). Most physical therapists had graduated from a vocational school (*n* = 1010, 74 %). Participants’ characteristics are summarized in Table 1.

**Table 1:** Demographic and work characteristics of the study participants (*n* = 1361)

Characteristic	n (%)
Age in years, mean (SD) range	41 (11) 20-73 *(n = 1330)*
Gender	
Female	839 (62)
Male	514 (38)
Other	3 (<1)
Missing	5 (<1)
Highest professional degree	
Diploma (vocational school)	1010 (74)
Bachelor/diploma (university)	246 (18)
Master	91 (7)
Doctorate	6 (<1)
Missing	8 (<1)
Work setting^a^	
Private practice	1191 (88)
Hospital	109 (8)
Rehabilitation clinic	71 (5)
Other	80 (6)
Missing	5 (<1)
Size of the city/municipality of employment	
Rural area (< 5000 inhabitants)	174 (13)
Small town (5000 – 20,000 inhabitants)	357 (26)
Mid-sized town (20,000 – 100,000 inhabitants)	338 (25)
Large town (> 100,000 inhabitants)	481 (35)
Missing	11 (1)
Number of newly admitted patients with LBP per week, median (25^th^-75^th^ percentile) range	2 (2-10) 0-20 *(n = 1195)*
Employment situation	
Employee/worker	792 (58)
Permanent employment	752 (95)
Fixed-term employment	38 (5)
Missing	2 (<1)
No. of colleagues, median (25^th^-75^th^ percentile) range	7 (4-11) 0-100 *(n = 778)*
Self-employment	473 (35)
No. of employees, median (25^th^-75^th^ percentile) range	3 (1-6) 0-60 *(n = 441)*
Freelancer	61 (5)
Missing	35 (3)
Work experience in years, mean (SD) range	16 (10) 1-45 *(n = 1338)*
Number of working hours per week, median (25^th^-75^th^ percentile) range	38 (30-40) 5-60 *(n = 1310)*
Further qualification relevant for LBP^a^	
Manual therapy	964 (71)
Orthopedic manual therapy	125 (9)
Healing practitioner	253 (19)
Osteopathy	191 (14)
Back school	461 (34)
Pain management physiotherapy	138 (10)
Other	545 (40)
None	158 (12)
Missing	17 (1)
Having inter-professional communication	
Yes	637 (47)
No	680 (50)
Missing	44 (3)

^a^ multiple answer possible; *LBP* Low back pain. Different sample size within each sample due to missing values

### Current clinical practice and guideline adherence

50 % (*n* = 675) of our respondents adhered to the recommendations of the NVL on the therapeutic diagnostic process, and 72 % (*n* = 973) to the recommendations concerning treatment. The guideline adherence across the entire management process (i.e., both diagnosis and treatment) of LBP was 38 % (*n* = 513).

#### Physical therapeutic diagnostic process

A total of 1321 (97 %) participants reported to conduct a physical therapeutic diagnostic process at first contact with patients with LBP, but there were differences in the level of detail within the examination. While 97 % (*n* = 1317) took medical history and asked for pain characteristics, only 79 % (*n* = 1068) included all characteristics of pain as recommended. The screening of all mentioned red flags, however, was only performed by 19 % (*n* = 253). The summary of all items mentioned in the scoring system is displayed in Table 2.

**Table 2:** Adherence to single items in the physical therapeutic diagnostic process of LBP

Item	n (%)	% of total study sample
Physical therapeutic diagnostic process	1321 (97)	97
Do you evaluate medical history?	1317 (100)	97
Do you ask about pain characteristics (localization, course, etc.)?	1317 (100)	97
Asking about all (6) pain characteristics	1068 (81)	79
Do you ask about extra-spinal causes?	947 (72)	70
Do you ask about ‘red flags’?	1161 (88)	85
Asking about all (5) ‘red flags’	253 (22)	19
Do you ask about ‘yellow flags’?	1022 (78)	75
Asking about ≥ 3 ‘yellow flags’	844 (83)	62
Do you ask about ‘black/blue flags’?	1154 (88)	85
Asking about ≥ 3 ‘black/blue flags’	1041 (90)	77
Do you conduct a physical examination (inspection, palpation, etc.)?	1320 (100)	97
Do you conduct an inspection?	1292 (98)	95
Do you conduct a palpation?	1275 (97)	94
Do you carry out an orienting movement testing?	1259 (95)	93
Do you test the straight leg raise and carry out the Bragard’s test in addition?	1119 (85)	82
Do you examine the sacroiliac joint?	1109 (84)	82
In case of suspected radicular symptoms, do you additionally conduct a neurological examination?	1029 (78)	83
Do you evaluate muscle strength to detect paresis?	911 (89)	67
Testing all (5) muscle groups	489 (54)	36
Do you test for sensory disorder?	846 (82)	62
Do you test the muscle reflexes?	520 (51)	38
Testing all (3) reflexes	296 (57)	22

#### Current physical therapy management

The three most common treatment choices for acute LBP were mobilisation of the lumbar spine (*n* = 1151, 85 %), heat therapy (*n* = 863, 63 %) and behavioural therapy (*n* = 736, 54 %). Massage (*n* = 626, 46 %) and Kinesio Taping (*n* = 390, 29 %) were often used despite being not recommended in the NVL.

**Fig. 1:**
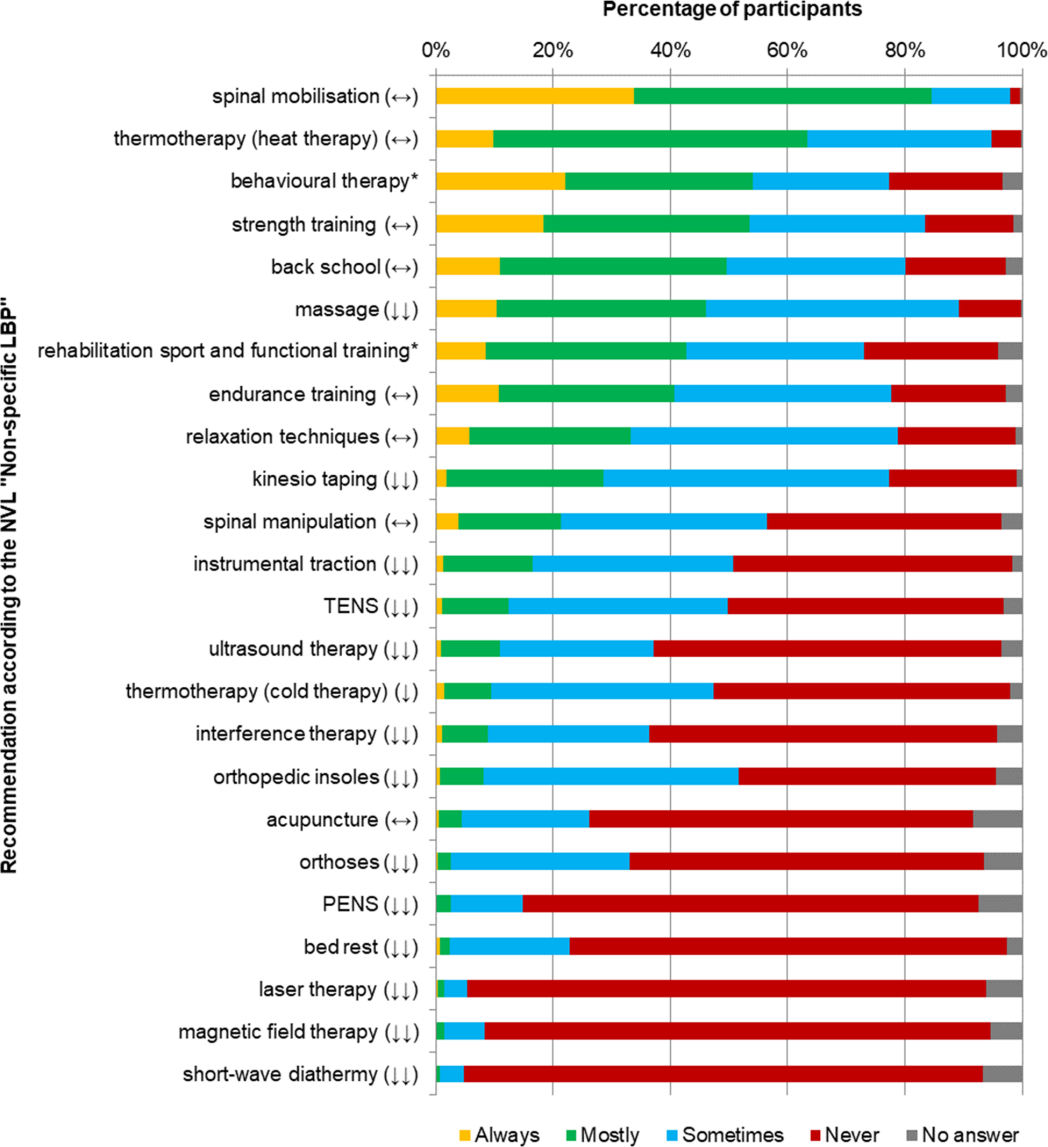
Application frequency of treatment modalities for acute LBP LBP: Low back pain; PENS: Percutaneous electrical nerve stimulation; TENS: Transcutaneous electrical nerve stimulation; “↑↑” = strong recommendation for a treatment; “↑”: recommendation for a treatment; “↔”: open recommendation; “↓”: recommendation against a treatment; “↓↓” = strong recommendation against a treatment; *no recommendation provided by the NVL

**Fig. 2:**
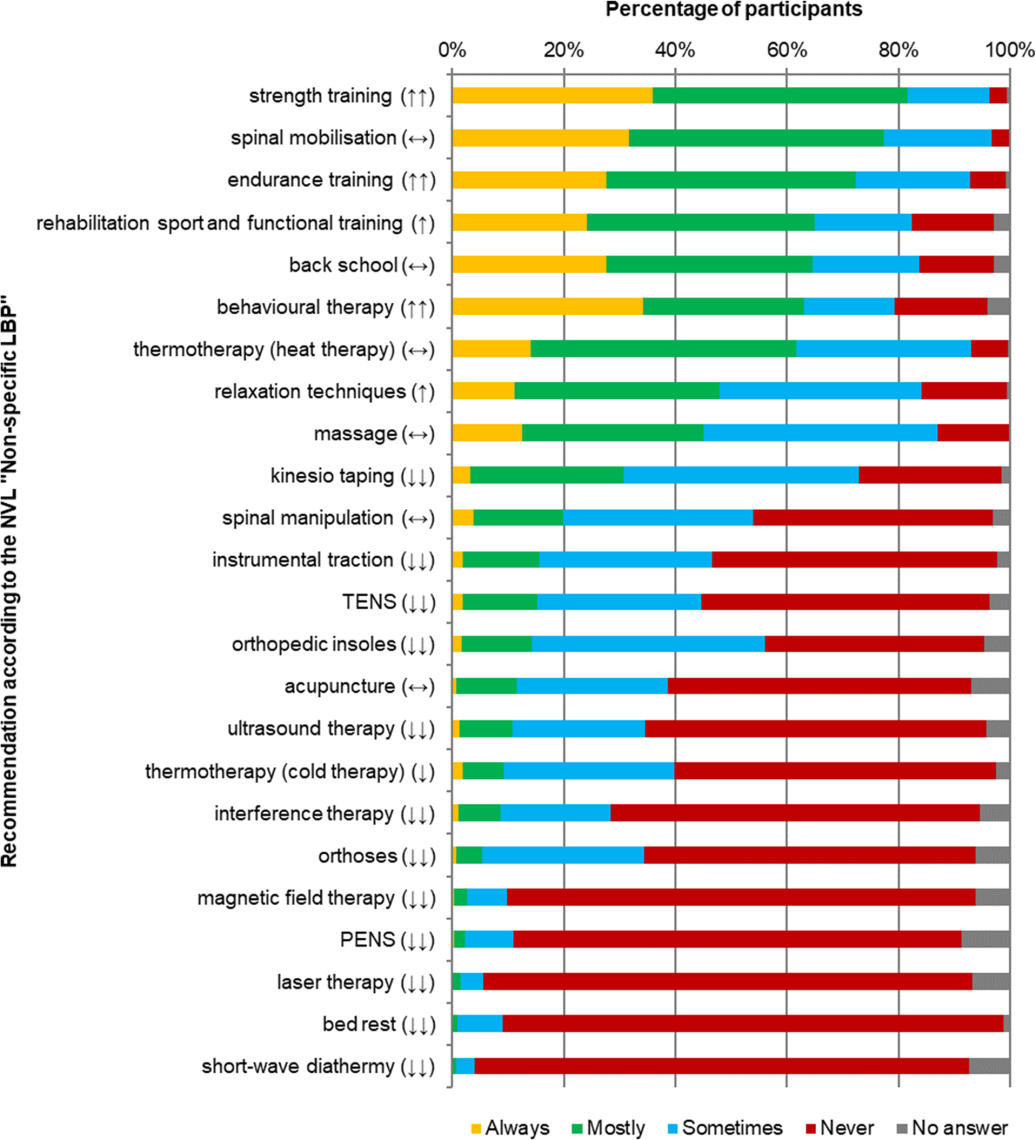
Application frequency of treatment modalities for chronic LBP LBP: Low back pain; PENS: Percutaneous electrical nerve stimulation; TENS: Transcutaneous electrical nerve stimulation; “↑↑” = strong recommendation for a treatment; “↑”: recommendation for a treatment; “↔”: open recommendation; “↓”: recommendation against a treatment; “↓↓” = strong recommendation against a treatment

The three most frequently applicated modalities in treating chronic LBP were strength training (*n* = 1110, 82 %), mobilisation of the lumbar spine (*n* = 1054, 77 %) and endurance training (*n* = 985, 72 %). With an application level of 31 % (*n* = 418) Kinesio Taping was the method most commonly used despite not being recommended. Figures 1 and 2 summarize the application frequency of different treatment modalities for acute and chronic LBP and their recommendation level according to the NVL.

### Determinants of guideline adherence

Physical therapists with bachelor or higher academic degree were more likely to show guideline adherence in the physical therapeutic diagnostic process (OR 1.71, 95 % CI 1.33–2.19), treatment process (OR 1.97, 95 % CI 1.47–2.68) and the entire management process (OR 1.93, 95 % CI 1.50–2.47) compared to physical therapists who received vocational education.

Univariate logistic regression analysis also identified sex and size of the city/municipality of employment as significantly associated with all models of guideline adherence (*p* < 0.05) (Table 3).

**Table 3:** Association between guideline adherence and demographic characteristics analyzed with univariate logistic regression model

	Guideline-adherent therapeutic diagnostic process	Guideline-adherent treatment	Total
		Odds ratio [95% CI]	
**Gender**^a^			
Female (*n* = 839)	Reference	Reference	Reference
Male (*n* = 514)	1.69 [1.36-2.12]*	1.66 [1.29-2.15]*	2.06 [1.64-2.58]*
**Highest professional degree**^a^			
Diploma (vocational school) (*n* = 1010)	Reference	Reference	Reference
Bachelor or higher academic degree (*n* = 343)	1.71 [1.33-2.19]*	1.97 [1.47-2.68]*	1.93 [1.50-2.47]*
**Work experience**^b^			
0-5 years (*n* = 243)	Reference	Reference	Reference
6-15 years (*n* = 482)	1.36 [1.00-1.85]*	0.81 [0.56-1.16]	1.32 [0.96-1.82]
16-25 years (*n* = 354)	1.31 [0.95-1.83]	0.80 [0.55-1.17]	1.21 [0.86-1.70]
26-35 years (*n* = 201)	1.19 [0.81-1.73]	0.54 [0.35-0.81]*	0.88 [0.59-1.31]
>35 years (*n* = 58)	1.46 [0.82-2.61]	0.40 [0.22-0.72]*	0.92 [0.49-1.68]
**Primary setting of work**^b^			
Private practice (*n* = 1107)	Reference	Reference	Reference
Hospital (*n* = 74)	1.09 [0.68-1.75]	0.77 [0.47-1.28]	0.89 [0.54-1.44]
Rehabilitation clinic (*n* = 52)	0.65 [0.36-1.14]	0.96 [0.53-1.83]	0.60 [0.31-1.10]
**Size of city/municipality of employment**^a^			
≥20,000 inhabitants (*n* = 819)	Reference	Reference	Reference
<20,000 inhabitants (*n* = 531)	0.71 [0.57-0.89]*	0.74 [0.58-0.94]*	0.70 [0.56-0.88]*
**Inter-professional collaboration**^a^			
No (*n* = 680)	Reference	Reference	Reference
Yes (*n* = 637)	1.76 [1.42-2.19]*	0.67 [0.52-0.85]*	1.25 [1.00-1.56]*

*LBP* Low back pain

* Significant, *p* < 0.05

^a^ OR calculated using a variable of the characteristic as reference

^b^ OR calculated as the ratio between the odds in the presence of characteristic variable against the odds in the absence of the variable

### Application of CPG and perceived barriers

Less than one third of study participants (*n* = 400, 29 %) indicated to know the NVL “Non-specific LBP” or had dealt with its recommendations, even though apart from the NVL 54 % (*n* = 733) stated to include guideline recommendations in clinical practice. Of the 35 % (*n* = 481) physical therapists who denied using guideline recommendations, almost 63 % (*n* = 304) were interested in doing so in the future. Table 4 shows perceived barriers to the application of guideline recommendations into clinical practice.

**Table 4:** Perceived barriers to the application of guideline recommendations (*n* = 481)

Perceived barrier	n (%)^**a**^
Guideline recommendations are not helpful to improve patient care	64 (13)
Guideline recommendations contradict my own clinical expertise	62 (13)
Using guidelines is not supported at my workplace	113 (24)
Guideline recommendations are not suitable given the individuality of the patients	259 (54)
Guideline recommendations are hindrances to my clinical decision-making	100 (21)
I do not have time to read guidelines	180 (37)
I do not know where I can find guidelines	121 (25)
Other	39 (8)

^a^Multiple answer possible

## Discussion

The objective of this study was to evaluate the current physical therapy management of patients with LBP in Germany, and to explore guideline adherence to the NVL “Non-specific LBP” recommendations. The results indicate that German physical therapists predominantly provide treatments recommended in the NVL guideline, but also frequently use treatments with low or conflicting evidence for patients with LBP. Even though guideline adherence in the treatment section was high, guideline adherence regarding the physical therapeutic diagnostic process seems deficient. In total, only 38 % of the participants showed guideline-adherent behaviour over both sections indicating deficits in evidence-based management in LBP in German physical therapists. With only 29 % of participants reporting to know the current version of the NVL “Non-specific LBP”, implementation of the NVL into physical therapy practice seems to have failed based on the results of this study.

Regarding the physical diagnostic process, about half of the items were identified by over 80 % of participants as a part of their physical therapeutic examination of patients with non-specific LBP. However, guideline adherence was fairly low (*n* = 675, 50 %). Screening red flags was performed by only 19 % (*n* = 253), despite being highly recommended in order to identify signs or symptoms indicating serious pathology requiring further medical diagnostics and different treatment [1, 20, 25]. However, as identified recently, consensus on red flags to be endorsed in guidelines is lacking [26]. Despite the recommendation in guidelines to screen red flags, there is evidence that only a few red flags are useful in clinical practice and the predictive power for serious diseases should be considered with caution. Screening of red flags is considered a necessary competence of physical therapists regarding direct access. Since direct access has not been established in the German health care system, this might partly explain the low guideline adherence in the physical therapeutic diagnostic process. Routinely, physical therapists are restricted to the physicians’ referral and diagnosis, and they lack time and reimbursement for a comprehensive examination of patients in routine care.

In line with the results from Basson et al. [27], mobilisation, heat application and exercise training were among the most frequently applied interventions by German physical therapists. Contrary to the results from previous studies, our results suggest that German physical therapists differentiate between the management of patients with acute and chronic LBP, as recommended in CPG. Notably in contrast to the findings from Zadro et al. [11], German physical therapists rarely use treatments that are not recommended. However, this might be distorted by the interprofessional scope of the NVL, which mentions several interventions less relevant in physical therapy context, hardly known or not feasible to be implemented in daily clinical practice, such as magnetic field therapy or laser therapy. Moreover, the low application rate of electrotherapy modalities, which are consistently not being recommended in the NVL, may be attributed to differences in the health care system of individual countries. While direct access has been implemented in the USA, Great Britain and Ireland, German physical therapists are bound to the physicians who must prescribe electrotherapy separately. German physicians most often prescribe “Krankengymnastik” [28] as the standard physical therapy treatment summarizing active and passive treatment techniques. In this case, physical therapists can apply different treatment modalities including both active and passive treatment approaches apart from special treatments such as electrotherapy. In the treatment of acute LBP, only massage, and in the treatment of both acute and chronic LBP, only Kinesio Taping were applied frequently despite having a negative recommendation.

The overall high guideline adherence in the treatment section (*n* = 973, 72 %) observed in our study was also found in a recent study of Danish physical therapists [29], where the vast majority of participants was strictly or partly in line with the CPG. In contrast to these results, de Souza et al. [30] stated that among Brazilian physical therapists, only 5–24 % showed full LBP guideline adherence. Ladeira et al. [31] reported 15–30 % adherence rate for physical therapists treating patients with acute LBP in Florida. Differences in reported guideline-adherent behaviour appear to be dependent upon several factors, including study design and the definition of guideline adherence. Husted et al. [29], de Souza et al. [30] and Ladeira et al. [31] evaluated guideline adherence using clinical vignettes. In contrast, participants of the current study ranked their application frequency of treatment modalities as recommended in the NVL and guideline adherence was determined using a scoring system. The benchmark for good adherence was set at ≥ 80 %, following the methods of a Belgian study [22] that measured physical therapists’ adherence to optimal knee osteoarthritis care. However, in our study, the benchmark refers to the point scores of each individual participant, whereas in Spitaels et al. [22] the 80 %-benchmark was used to describe good adherence within each quality indicator regarding the total sample.

In line with previous findings [23], the results of our study indicate greater guideline adherence of physical therapists with academic background (clinical examination: OR 1.71, 95 % CI 1.33–2.19; treatment: OR 1.97, 95 % CI 1.47–2.68; total: OR 1.93, 95 % CI 1.50–2.47). Therapists who deal with scientific literature and have scientific understanding use evidence-based practice (EBP) more frequently [32]. However, due to the exploratory nature of our analyses, ORs should be interpreted with caution.

Our results indicate that the utility of the NVL “Non-specific LBP” as a CPG for German physical therapists should be discussed. Even though the NVL intends to be a decision-making aid for both physicians and non-medical professionals, the guideline contains little specific information for physical therapists. While this may partly be attributable to a lack of scientific evidence, guidelines specific to physical therapy do exist in other countries such as the US [33] or the Netherlands [20]. Obviously, factors such as the involvement of disciplines and authors, the guideline topic, or the health policy of the respective country can influence the contents and recommendations of CPGs [21, 34]. Although physical therapy representatives were involved in the German NVL development, the percentage from physical therapist in relation to all stakeholders was low.

More than half of participants reported to include guideline recommendations within clinical practice, but only 29 % of the therapists (*n* = 400) responded that they knew the current version of the NVL “non-specific LBP”. Of those therapists who did not yet follow evidence-based recommendations, 63 % (*n* = 304) stated that they had a general interest in using guidelines. Thus, improving implementation strategies for guidelines into physical therapy practice seems to offer vast potential. An important prerequisite for improving implementation strategies is the identification of barriers in the application of guideline recommendations [35].

In line with previous studies [13, 36–38], survey participants reported inapplicability of guideline recommendations to individual patients (*n* = 259, 54 %), time restrictions (*n* = 180, 37 %) and lack of research skills (*n* = 121, 25 %) as the most important factors inhibiting the use of guidelines in clinical practice. In a Danish study [29], the authors assumed an association between increased guideline adherence and more time spend on the first consultation (60 min or more). A typical physical therapy session in Germany lasts about 15–20 min for patients with musculoskeletal disorders, which poses an important structural barrier. Therapists may need to treat about 20 patients a day, which makes it nearly impossible to search and critically appraise the current evidence [39].

Extensive EBP implementation interventions with frequent contacts have been shown to be more successful in changing the clinical behaviour of health care practitioners and improving patient outcomes than single or one-off interventions [40]. There is still no established approach or framework for transferring EBP into the German health care system [41]. Attitudes and beliefs towards EBP may heavily influence the clinical practice of therapists [42]. Thus, in addition to the removal of existing structural barriers, a successful implementation of research findings may require a change in the attitude and behaviour of physical therapists [25]. This may be a very challenging and ambitious goal, but better adherence to guideline recommendations may considerably improve patient outcomes and reduce health care costs in patients with LBP [12, 43]. Greater utilization of EBP should be of interest to all stakeholders.

### Limitations

Although we tried to distribute the survey to a broad number of physical therapists using the snowball sampling approach, the number of study participants (*n* = 1361) was low compared to the total number of approximately 199,000 physical therapists [44] working in Germany. However, because the exact number of physical therapists remains unclear and there is no information on how many physical therapists manage patients with LBP, no exact response rate could be calculated. The targeted sample size of at least 1000 participants was achieved, but physical therapists with an academic education (25 %) were overrepresented in our sample compared to their assumed number in Germany (3 %) [24].

Using an online survey may have introduced bias by possibly excluding physical therapists without internet access or online content proficiency. Furthermore, it could not be definitively ascertained whether the participants actually met the inclusion criteria, or whether they had participated more than once, as the survey was anonymous and accessible without legitimation. Participants may have looked up the guideline after completing the questionnaire for the first time, which may have influenced our findings towards higher guideline adherence with their second participation. Duplicate responses could have been prevented using a cookie- or IP-based duplicate protection, but this would also have limited study participation via shared devices (for example used in physical therapy facilities) and would have reduced the number of participants.

The data reflect what participants reported, as opposed to how they actually perform their examination and treatment of patients with non-specific LBP. Our study results may be influenced towards higher guideline adherence, as due to social desirability, recommended behaviour is usually being over-reported, and behaviour contrary to guideline recommendations under-reported. In a systematic review, Adams et al. [45] determined that guideline adherence assessed through self-report measures was over-estimated by about 27 % compared to objective methods. Further, sampling bias due to the overrepresentation of physical therapists with an academic background and volunteer bias must be assumed. Therapists with a personal interest in LBP might have participated more readily and might have better knowledge of LBP management than non-respondents.

No firm conclusions on the actual quality of physical therapy management can be drawn. For example, the intensity, frequency and duration of strength training may determine its effectiveness, but the NVL lacks any such information. Thus, these aspects were not evaluated in this study.

Although advice and education are internationally stated to be important aspects of physical therapy management in LBP [27], these interventions were not evaluated in our study, because advice and education should primarily be provided by physicians in Germany. Advice/education is not addressed in the German catalogue of therapies [10] and physical therapists are not formally educated accordingly. However, as time per patient is also limited for physicians, it seems reasonable to assume that physical therapists provide advice and education. Future studies should therefore evaluate the current clinical practice regarding advice/education as well as the content of possible recommendations provided by physical therapists in the management of LBP.

## Conclusions

Based on the results of this study, guideline adherence in the management of patients with LBP by German physical therapists offers potential for enhancement, especially regarding the physical therapeutic diagnostic process. Although the NVL “Non-specific LBP” intends to be a CPG for both physicians and other health care professionals, the proportion of physical therapists who know of the NVL was low. Reduced applicability of guideline recommendations to individual patients and structural barriers were mentioned as the most important factors inhibiting the use of guidelines in clinical practice. Improved implementation strategies and the removal of existing barriers against the application of evidence-based guidelines may facilitate the transfer of evidence into clinical practice and contribute to optimized quality of health care.

## Supplementary Information

**Additional file 1.** Complete online survey (in German language)

**Additional file 2.** Translated English language version of the questionnaire

## Data Availability

The datasets used and/or analysed during the current study are available from the corresponding author on reasonable request.

## Abbreviations

CHERRIES: Checklist for Reporting Results of Internet E-Surveys
CPG: Clinical practice guideline
EBP: Evidence-based practice
KNGF: Royal Dutch Society for Physical Therapy
LBP: Low back pain
NVL: National Disease Management Guideline
TENS: Transcutaneous electrical nerve stimulation

## References

[1] Bundesärztekammer, Kassenärztliche Bundesvereinigung, Arbeitsgemeinschaft der Wissenschaftlichen Medizinischen Fachgesellschaften. Nationale Versorgungs-Leitlinie Nicht-spezifischer Kreuzschmerz - Langfassung, 2. Auflage. Version 1. 2017.

[2] van Tulder M, Becker A, Bekkering T, Breen A, del Real MT, Hutchinson A (2006). Chapter 3. European guidelines for the management of acute nonspecific low back pain in primary care. Eur Spine J.

[3] Hartvigsen J, Hancock MJ, Kongsted A, Louw Q, Ferreira ML, Genevay S (2018). What low back pain is and why we need to pay attention. Lancet.

[4] Hestbaek L, Leboeuf-Yde C, Manniche C (2003). Low back pain: what is the long-term course? A review of studies of general patient populations. Eur Spine J.

[5] Hoy D, March L, Brooks P, Blyth F, Woolf A, Bain C (2014). The global burden of low back pain: estimates from the Global Burden of Disease 2010 study. Ann Rheum Dis.

[6] Lidgren L (2003). The bone and joint decade 2000–2010. Bull World Health Organ.

[7] Murray CJ, Vos T, Lozano R, Naghavi M, Flaxman AD, Michaud C (2012). Disability-adjusted life years (DALYs) for 291 diseases and injuries in 21 regions, 1990–2010: a systematic analysis for the Global Burden of Disease Study 2010. Lancet.

[8] Dagenais S, Caro J, Haldeman S (2008). A systematic review of low back pain cost of illness studies in the United States and internationally. Spine J.

[9] Foster NE, Anema JR, Cherkin D, Chou R, Cohen SP, Gross DP (2018). Prevention and treatment of low back pain: evidence, challenges, and promising directions. Lancet.

[10] IntelliMed GmbH. Heilmittelkatalog 2018. www.heilmittelkatalog.de. Accessed 09 Apr 2021.

[11] Zadro J, O’Keeffe M, Maher C (2019). Do physical therapists follow evidence-based guidelines when managing musculoskeletal conditions? Systematic review. BMJ Open.

[12] Hanney WJ, Masaracchio M, Liu X, Kolber MJ (2016). The Influence of Physical Therapy Guideline Adherence on Healthcare Utilization and Costs among Patients with Low Back Pain: A Systematic Review of the Literature. PLoS One.

[13] Bernhardsson S, Johansson K, Nilsen P, Öberg B, Larsson ME (2014). Determinants of guideline use in primary care physical therapy: a cross-sectional survey of attitudes, knowledge, and behavior. Phys Ther.

[14] Deitermann B, Kemper C, Hoffmann F, Glaeske G. GEK-Heil- und Hilfsmittel-Report 2006: Auswertungsergebnisse der GEK-Heil- und Hilfsmitteldaten aus den Jahren 2004 und 2005. GEK Edition. St. Augustin: Asgard-Verlag; 2006.

[15] Kemper C, Sauer K, Glaeske G. BARMER GEK-Heil- und Hilfsmittel-Report 2011: Auswertungsergebnisse der BARMER GEK-Heil- und Hilfsmitteldaten aus den Jahren 2009 und 2010. BARMER GEK Edition. St. Augustin: Asgard-Verlag; 2011.

[16] Konrad R, Konrad A, Geraedts M. Ausbildung von Physiotherapeutinnen und Physiotherapeuten in Deutschland: Bereit für den Direktzugang? Gesundheitswesen. 2017;79(07):e48-e55. 10.1055/s-0035-1559708 26406769

[17] Dreier G, Hasselblatt H, Antes G, Schumacher M (2009). Das Deutsche Register Klinischer Studien: Begründung, technische und inhaltliche Aspekte, internationale Einbindung. Bundesgesundheitsblatt Gesundheitsforschung Gesundheitsschutz.

[18] Eysenbach G (2004). Improving the quality of Web surveys: the Checklist for Reporting Results of Internet E-Surveys (CHERRIES). J Med Internet Res.

[19] National Guideline Centre. National Institute for Health and Care Excellence (2016). Clinical Guidelines. Low Back Pain and Sciatica in Over 16s: Assessment and Management.

[20] Royal Dutch Society for Physical Therapy (Koninklijk Nederlands Genootschap voor Fysiotherapie KNGF). KNGF Clinical Practice Guideline for Physical Therapy in patients with low back pain. 2013.

[21] da Silva TM, Costa Lda C, Garcia AN, Costa LO (2015). What do physical therapists think about evidence-based practice? A systematic review. Man Ther.

[22] Spitaels D, Hermens R, Van Assche D, Verschueren S, Luyten F, Vankrunkelsven P (2017). Are physiotherapists adhering to quality indicators for the management of knee osteoarthritis? An observational study. Musculoskelet Sci Pract.

[23] Hendrick P, Mani R, Bishop A, Milosavljevic S, Schneiders AG (2013). Therapist knowledge, adherence and use of low back pain guidelines to inform clinical decisions–a national survey of manipulative and sports physiotherapists in New Zealand. Man Ther.

[24] PhysioDeutschland Deutscher Verband für Physiotherapie (ZVK) e.V. Zahlen, Daten, Fakten zur Physiotherapie. 2021. https://www.physio-deutschland.de/fileadmin/data/bund/Dateien_oeffentlich/Beruf_und_Bildung/Zahlen__Daten__Fakten/Zahlen-Daten-Fakten-Jan21.pdf. Accessed 04 Apr 2021.

[25] O’Connell NE, Ward SP (2018). Low Back Pain: What Have Clinical Guidelines Ever Done for Us?. J Orthop Sports Phys Ther.

[26] Verhagen AP, Downie A, Popal N, Maher C, Koes BW (2016). Red flags presented in current low back pain guidelines: a review. Eur Spine J.

[27] Basson A. Physiotherapy management of low back pain - a review of surveys. South African Journal of Physiotherapy; Vol 67, No 1 (2011). 2011.

[28] Kopkow C, Lange T, Schmitt J, Petzold T (2017). [Utilization of Physical Therapy Services in Germany from 2004 until 2014: Analysis of Statutory Health Insurance Data]. Gesundheitswesen.

[29] Husted M, Rossen CB, Jensen TS, Mikkelsen LR, Rolving N (2020). Adherence to key domains in low back pain guidelines: A cross-sectional study of Danish physiotherapists. Physiother Res Int.

[30] de Souza FS, Ladeira CE, Costa LOP (2017). Adherence to Back Pain Clinical Practice Guidelines by Brazilian Physical Therapists: A Cross-sectional Study. Spine (Phila Pa 1976).

[31] Ladeira CE, Samuel Cheng M, Hill CJ (2015). Physical therapists’ treatment choices for non-specific low back pain in Florida: an electronic survey. J Man Manip Ther.

[32] Iles R, Davidson M (2006). Evidence based practice: a survey of physiotherapists’ current practice. Physiother Res Int.

[33] Delitto A, George SZ, Van Dillen L, Whitman JM, Sowa G, Shekelle P (2012). Low back pain. The Journal of orthopaedic and sports physical therapy.

[34] Peter WF, van der Wees PJ, Verhoef J, de Jong Z, van Bodegom-Vos L, Hilberdink WK (2013). Postgraduate education to increase adherence to a Dutch physiotherapy practice guideline for hip and knee OA: a randomized controlled trial. Rheumatology (Oxford).

[35] Cote AM, Durand MJ, Tousignant M, Poitras S (2009). Physiotherapists and use of low back pain guidelines: a qualitative study of the barriers and facilitators. J Occup Rehabil.

[36] Heiwe S, Kajermo KN, Tyni-Lenne R, Guidetti S, Samuelsson M, Andersson IL (2011). Evidence-based practice: attitudes, knowledge and behaviour among allied health care professionals. Int J Qual Health Care.

[37] Jette DU, Bacon K, Batty C, Carlson M, Ferland A, Hemingway RD (2003). Evidence-based practice: beliefs, attitudes, knowledge, and behaviors of physical therapists. Phys Ther.

[38] Upton D, Upton P (2006). Knowledge and use of evidence-based practice by allied health and health science professionals in the United Kingdom. J Allied Health.

[39] Spitzenverband Bund der Krankenkassen. Anlage 1a zu den Rahmenempfehlungen nach § 125 Abs. 1 SGB V vom 1. August 2001 in der Fassung vom 1. Juni 2006 Leistungsbeschreibung Physiotherapie. 2006. https://www.vdek.com/vertragspartner/heilmittel/rahmenempfehlung/_jcr_content/par/download_0/file.res/re_125_anl_1a_2010.pdf. Accessed 18 Feb 2018.

[40] Mesner SA, Foster NE, French SD (2016). Implementation interventions to improve the management of non-specific low back pain: a systematic review. BMC Musculoskelet Disord..

[41] Institut für Qualität und Wirtschaftlichkeit im Gesundheitswesen. Umsetzung von Leitlinien – hinderliche und förderliche Faktoren. 2016. https://www.iqwig.de/de/projekte-ergebnisse/projekte/versorgung/v12-04-umsetzung-von-leitlinien-hinderliche-und-foerderliche-faktoren.6922.html. Accessed 18 Feb 2018.

[42] Gardner T, Refshauge K, Smith L, McAuley J, Hübscher M, Goodall S. Physiotherapists’ beliefs and attitudes influence clinical practice in chronic low back pain: A systematic review of quantitative and qualitative studies. J Physiother. 2017;63. 10.1016/j.jphys.2017.05.017 28655562

[43] Fritz JM, Cleland JA, Brennan GP (2007). Does adherence to the guideline recommendation for active treatments improve the quality of care for patients with acute low back pain delivered by physical therapists?. Med Care.

[44] World Physiotherapy. German Association for Physiotherapy. 2021. https://world.physio/membership/germany. Accessed 07 Apr 2021.

[45] Adams AS, Soumerai SB, Lomas J, Ross-Degnan D (1999). Evidence of self-report bias in assessing adherence to guidelines. Int J Qual Health Care.

