# Supplementary material for: Physical therapy for patients with low back pain in Germany: a survey of current practice
Source: BMC Musculoskelet Disord. 2021 Jun 19;22:563. doi: 10.1186/s12891-021-04422-2 (PMC8214788; doi:10.1186/s12891-021-04422-2)
Supplement: Supplementary file 1 — Additional file 1. Complete online survey (in German language) [file 12891_2021_4422_MOESM1_ESM.pdf]

hsg Hochschule für Gesundheit

0% ausgefüllt

Sehr geehrte Teilnehmerin, sehr geehrter Teilnehmer,

vielen Dank, dass Sie sich für unsere Online-Befragung zum Thema „Physiotherapeutische Versorgungssituation von Patienten*innen mit Kreuzschmerz in Deutschland" interessieren.

Was wird untersucht?

Wir würden Ihnen gerne einige Fragen zu den folgenden vier Themenbereichen stellen:

1. Allgemeine demografische und berufliche Angaben
2. Inhalte und Umfang des therapeutischen Befundes bei Patienten*innen mit akuten und chronischen Kreuzschmerzen
3. Inhalte und Empfehlungen zur Behandlung von Patienten*innen mit akuten und chronischen Kreuzschmerzen
4. Kenntnis und Anwendung von wissenschaftlichen Leitlinien in der beruflichen Tätigkeit als Physiotherapeut*in

Was ist das Ziel der Studie?

Ziel der Studie ist die Erfassung der aktuellen Versorgungssituation im physiotherapeutischen Kontext von Patienten*innen mit Rückenschmerzen in Deutschland bezüglich der Empfehlungen der Nationalen Versorgungsleitlinie (NVL) "Nicht-spezifischer Kreuzschmerz", 2. Auflage.

Wie zeitaufwändig ist die Teilnahme?

Die Teilnahme an der Online-Befragung nimmt ca. **15 Minuten** in Anspruch.

Was passiert mit meinen Daten?

Die erhobenen Daten werden unter Verschluss gehalten und nur in anonymisierter Form zu wissenschaftlichen Zwecken veröffentlicht.

Welche Risiken gibt es?

Die Befragung ist mit keinen Risiken verbunden.

Ihre Teilnahme an dieser Studie erfolgt freiwillig. Sie können jederzeit ohne Angabe von Gründen Ihre Teilnahme zurückziehen, ohne dass Ihnen dadurch Nachteile entstehen. Sie sind nicht dazu verpflichtet jede Frage zu beantworten und können die Befragung

jederzeit beenden.

Datenschutzerklärung

§ Ich willige ein, dass im Rahmen der Studie erhobene Daten auf elektronischen Datenträgern aufgezeichnet und in pseudonymisierter* Form bei dem Erhebungs- und Auswertungszentrum** der Studie zur wissenschaftlichen Auswertung gespeichert werden.

§ Außerdem willige ich ein, dass ein autorisierter und zur Verschwiegenheit verpflichteter Beauftragter der Ethik-Kommission in meine beim Erhebungs- und Auswertungszentrum** vorhandenen personenbezogenen Daten Einsicht nehmen darf, soweit dies für die Überprüfung der Studie notwendig ist. Für diese Maßnahme entbinde ich das Erhebungs- und Auswertungszentrum von der Schweigepflicht.

§ Ich weiß, dass ich meine Zustimmung jederzeit widerrufen kann, ohne dass mir daraus Nachteile entstehen.

* Pseudonymisieren ist das Ersetzen des Namens und anderer Identifikationsmerkmale durch ein Kennzeichen zu dem Zweck, die Bestimmung des Betroffenen auszuschließen oder wesentlich zu erschweren (§ 3 Abs. 6a BDSG).

** Prof. Dr.Christian Kopkow, Hochschule für Gesundheit Bochum, Department für Angewandte Gesundheitswissenschaften, Gesundheitscampus 6-8, 44801 Bochum

Ich habe die Teilnehmerinformation zur Studie "Physiotherapeutische Versorgungssituation von Patienten*innen mit Kreuzschmerz in Deutschland" verstanden und willige der Studienteilnahme sowie der pseudonymisierten Aufzeichnung und Auswertung meiner Daten, deren Weitergabe und der Einsichtnahme in meine personenbezogenen Daten in der oben beschriebenen Form ein.

Sie müssen auf „**Weiter**“ klicken, um an der Umfrage teilnehmen zu dürfen.

Weiter

Hochschule für Gesundheit

3% ausgefüllt

Hinweise zur Beantwortung der Fragen

Beachten Sie bitte, dass die Befragung an einem Stück beantwortet werden muss, um den Verlust von Daten zu verhindern (**Zwischenspeichern von Ergebnissen ist nicht möglich**). Zudem möchten wir Sie darauf hinweisen, dass Sie, sobald Sie auf „Weiter" klicken, **nicht** zur vorherigen Seite der Befragung zurück wechseln können.

Daher nehmen Sie sich bitte ausreichend Zeit, um die folgenden Fragen zu lesen und nach bestem Wissen und Gewissen zu beantworten.

Vielen Dank für Ihre Teilnahme an dieser wichtigen Studie!

Weiter

hsg Hochschule für Gesundheit

6% ausgefüllt

I. Allgemeine Angaben

1. Wie alt sind Sie?

Jahre | ☐ keine Angabe

2. Welches Geschlecht haben Sie?

○ männlich ○ weiblich ○ andere | ○ keine Angabe

3. In was für einer Einrichtung sind Sie tätig?

Mehrfachnennungen möglich

☐ freie Praxis
☐ Krankenhaus
☐ Rehabilitationsklinik
☐ Sonstiges:

☐ keine Angabe

4. In welchem Bundesland arbeiten Sie?

[Bitte auswählen]

5. Wie viele Einwohner leben in der Stadt (oder der Gemeinde), in welcher sich Ihr Arbeitsplatz befindet?
Wenn Sie mehrere Arbeitsplätze haben, dann beantworten Sie diese Frage bitte in Bezug auf den Arbeitsplatz, an welchem Sie die meiste Arbeitszeit tätig sind.

○ <5.000 Einwohner (Landgemeinde)
○ 5.000-20.000 Einwohner (Kleinstadt)
○ 20.000-100.000 Einwohner (Mittelstadt)
○ >100.000 Einwohner (Großstadt)

○ keine Angabe

6. Sind Sie:

*Wenn Sie mehrere Arbeitsstellen als Physiotherapeut*in haben, dann beantworten Sie diese Frage bitte in Bezug auf ihr Hauptarbeitsverhältnis.*

○	○	○	○
Arbeitnehmer	selbstständig	freiberuflich	keine Angabe

Weiter

Questions 7 and 8 only appear if question 6 (screen 3) was answered with "Arbeitnehmer".

hsg Hochschule für Gesundheit

9% ausgefüllt

7. Wie ist Ihr aktuelles Anstellungsverhältnis?

○ unbefristete Anstellung
○ befristete Anstellung
○ keine Angabe

8. Wie viele Kollegen*innen arbeiten in Ihrem Team?
*(Nur Physiotherapeuten*innen)*

Kollegen*innen
☐ keine Angabe

9. Wie viele Stunden pro Woche sind Sie als Physiotherapeut*in tätig?

Stunden/Woche
☐ keine Angabe

10. Wo haben Sie Ihre berufliche Ausbildung absolviert?

○ Berufsfachschule
○ Hochschule
○ Sonstiges (z.B. Hochschule im Ausland, Duales Studium, ...)

○ keine Angabe

11. Welche/n der folgenden Abschlüsse haben Sie bereits im gesundheitlichen Fachbereich erworben?

Mehrfachnennungen möglich

☐ staatlich anerkannter/e Physiotherapeut*in
☐ Diplom
☐ Bachelor
☐ Master
☐ Doktorgrad

☐ keine Angabe

12. Wie lange arbeiten Sie bereits als examinierter Physiotherapeut*in?

Jahr(e)
☐ keine Angabe

13. Wie viele neu aufgenommene Patienten*innen mit Kreuzschmerzen behandeln Sie typischerweise innerhalb einer Arbeitswoche?

Patienten*innen pro Woche

☐ keine Angabe

14. Haben Sie eine oder mehrere der folgenden Fortbildungen abgeschlossen?

Mehrfachnennungen möglich

☐ Manuelle Therapie (MT)
☐ Orthopädisch manipulative Therapie (OMT)
☐ Heilpraktiker
☐ Osteopathie
☐ Rückenschule
☐ Schmerzphysiotherapie
Weitere:
☐
(Bitte nur für Kreuzschmerz relevante Fortbildungen angeben.)
☐ keine

☐ keine Angabe

15. Befinden Sie sich in Ihrer Einrichtung im direkten Austausch mit anderen Gesundheitsberufen (Ärzte, Psychologen, Ergotherapeuten, ...)?

○ Ja
○ Nein
○ keine Angabe

Weiter

Question 7 only appears if question 6 (screen 3) was answered with "selbstständig".

hsg Hochschule für Gesundheit

9% ausgefüllt

7. Wie viele Mitarbeiter*innen arbeiten in Ihrem Team?

____ Mitarbeiter*innen | ☐ keine Angabe

8. Wie viele Stunden pro Woche sind Sie als Physiotherapeut*in tätig?

____ Stunden/Woche | ☐ keine Angabe

9. Wo haben Sie Ihre berufliche Ausbildung absolviert?

○ Berufsfachschule
○ Hochschule
○ Sonstiges (z.B. Hochschule im Ausland, Duales Studium, ...)

○ keine Angabe

10. Welche/n der folgenden Abschlüsse haben Sie bereits im gesundheitlichen Fachbereich erworben?

Mehrfachnennungen möglich

☐ staatlich anerkannter/e Physiotherapeut*in
☐ Diplom
☐ Bachelor
☐ Master
☐ Doktorgrad

☐ keine Angabe

11. Wie lange arbeiten Sie bereits als examinierter Physiotherapeut*in?

____ Jahr(e) | ☐ keine Angabe

12. Wie viele <u>neu aufgenommene</u> Patienten*innen mit Kreuzschmerzen behandeln Sie typischerweise innerhalb einer Arbeitswoche?

____ Patienten*innen pro Woche | ☐ keine Angabe

13. Haben Sie eine oder mehrere der folgenden Fortbildungen abgeschlossen?

Mehrfachnennungen möglich

- ☐ Manuelle Therapie (MT)
- ☐ Orthopädisch manipulative Therapie (OMT)
- ☐ Heilpraktiker
- ☐ Osteopathie
- ☐ Rückenschule
- ☐ Schmerzphysiotherapie
- ☐ Weitere: ____________________
  (Bitte nur für Kreuzschmerz relevante Fortbildungen angeben.)
- ☐ keine

- ☐ keine Angabe

14. Befinden Sie sich in Ihrer Einrichtung im direkten Austausch mit anderen Gesundheitsberufen (Ärzte, Psychologen, Ergotherapeuten, ...)?

○	○	○
Ja	Nein	keine Angabe

Weiter

Question 16 only appears if question 15 (screen 4) was answered with "Ja".

12% ausgefüllt

16. Mit welchen Berufsgruppen befinden Sie sich an Ihrer Arbeitsstelle im Austausch?

Mehrfachnennungen möglich

- ☐ Ärzte
- ☐ Psychotherapeuten
- ☐ Pflegekräfte
- ☐ Ergotherapeuten
- ☐ Sonstige: ______

- ☐ keine Angabe

17. Wie viel Zeit steht Ihnen im Rahmen eines normalen Arbeitstages jeweils für folgende Tätigkeiten zur Verfügung?

Erstbefund:	____ min	☐ keine Angabe
Behandlung:	____ min	☐ keine Angabe

Weiter

Hochschule für Gesundheit

15% ausgefüllt

II. Therapeutischer Befund

18. Unterscheiden Sie zwischen akuten und chronischen Rückenschmerzen?

Unter akuten Kreuzschmerzen werden neu aufgetretene Schmerzepisoden, die weniger als sechs Wochen anhalten, zusammengefasst. Chronische Kreuzschmerzen werden als Kreuzschmerzen, die länger als zwölf Wochen anhalten, bezeichnet.

○ Ja

○ Nein

○ keine Angabe

19. Führen Sie einen therapeutischen Erstbefund durch?

○ Ja

○ Nein

○ keine Angabe

Weiter

Question 20 only appears if question 19 (screen 6) was answered with "Ja".

18% ausgefüllt

20. Führen Sie eine Anamnese durch?

- ◯ Ja
- ◯ Nein

- ◯ keine Angabe

Weiter

Question 21 only appears if question 20 (screen 7) was answered with "Ja".

Hochschule für Gesundheit

21% ausgefüllt

21. Erfragen Sie Schmerzeigenschaften (z.B. Lokalisation, Beginn, Verlauf, ...)?

- ○ Ja
- ○ Nein

- ○ keine Angabe

Weiter

Question 22 only appears if question 21 (screen 8) was answered with "Ja".

24% ausgefüllt

22. Welche Eigenschaften erfragen Sie?

Mehrfachnennungen möglich

- ☐ Lokalisation und Ausstrahlung
- ☐ Beginn
- ☐ auslösende, verstärkende oder lindernde Faktoren
- ☐ (tages-) zeitlicher Verlauf
- ☐ Stärke und Beeinträchtigung bei täglichen Verrichtungen
- ☐ frühere Episoden

- ☐ keine Angabe

Weiter

Question 23 only appears if question 20 (screen 7) was answered with "Ja".

Hochschule für Gesundheit

27% ausgefüllt

23. Erfragen Sie extravertebragene Ursachen?

Extravertebragene Ursachen sind definiert als Schmerzen im Bereich der Lendenwirbelsäule, die durch benachbarte Organe ausgelöst werden, die nicht unmittelbar zu den knöchernen, muskulären oder diskoligamentären Strukturen der Wirbelsäule gehören (z.B. durch ein Aortenaneurysma).

- ○ Ja
- ○ Nein

- ○ keine Angabe

Weiter

Question 24 only appears if question 23 (screen 10) was answered with "Ja".

30% ausgefüllt

24. Welche extravertebragenen Ursachen erfragen Sie?

Mehrfachnennungen möglich

- ☐ abdominelle und viszerale Prozesse (z.B. Pankreatitis)
- ☐ Gefäßveränderungen (z.B. Aortenaneurysmen)
- ☐ gynäkologische Ursachen (z.B. Endometriose)
- ☐ urologische Ursachen (z.B. Urolitiasis)
- ☐ neurologische Erkrankungen (z.B. Polyneuropathie)
- ☐ psychosomatische und psychiatrische Erkrankungen

- ☐ keine Angabe

Weiter

Questions 25, 26 and 27 only appear if question 20 (screen 7) was answered with "Ja".

hsg Hochschule für Gesundheit

33% ausgefüllt

25. Erfragen Sie „Red Flags"?

*„Red Flags" sind definiert als Warnhinweise für eine spezifische Ursache der Kreuzschmerzen, die eine kurzfristige und gegebenenfalls notfallmäßige Abklärung und Therapie durch einen/e Arzt*in erfordern.*

- ○ Ja
- ○ Nein

- ○ keine Angabe

26. Erfragen Sie „Yellow Flags"?

„Yellow Flags" sind definiert als psychosoziale Faktoren, die das Risiko für eine Chronifizierung von Kreuzschmerzen erhöhen und für den Krankheitsverlauf eine entscheidende Rolle spielen.

- ○ Ja
- ○ Nein

- ○ keine Angabe

27. Erfragen Sie „Blue" und „Black Flags"?

„Blue/Black Flags" sind definiert als arbeitsplatzbezogene Faktoren, die das Entstehen chronischer Kreuzschmerzen beeinflussen.

- ○ Ja
- ○ Nein

- ○ keine Angabe

Weiter

Questions 28, 29 and 30 only appear if questions 25, 26, 27 (screen 12) were answered with "Ja".

hsg Hochschule für Gesundheit

36% ausgefüllt

28. Für welche der folgenden Pathologien erfragen Sie „Red Flags“?

Mehrfachnennungen möglich

- ☐ Fraktur/Osteoporose
- ☐ Infektion
- ☐ Radikulopathien/Neuropathien
- ☐ Tumor/Metastasen
- ☐ axiale Spondyloarthritis

- ☐ keine Angabe

29. Welche „Yellow Flags“ erfragen Sie?

Mehrfachnennungen möglich

- ☐ Depressivität/Distress (negativer Stress)
- ☐ schmerzbezogene Kognitionen (z.B. Angstvermeidungsverhalten,Katastrophisieren)
- ☐ passives Schmerzverhalten (z.B. ausgeprägtes Schon- und Angstvermeidungsverhalten)
- ☐ überaktives Schmerzverhalten (beharrliche Arbeitsamkeit)
- ☐ schmerzbezogene Kognitionen (Gedankenunterdrückung)
- ☐ Neigung zur Somatisierung (Auftreten körperlicher Beschwerden, die keine organische Ursache haben, sondern psychisch bedingt sind)

- ☐ keine Angabe

30. Welche „Blue“ und „Black Flags“ erfragen Sie?

Mehrfachnennungen möglich

- ☐ überwiegend körperliche Schwerarbeit
- ☐ überwiegend monotone Körperhaltung
- ☐ überwiegend Vibrationsexposition
- ☐ geringe berufliche Qualifikation
- ☐ geringer Einfluss auf die Arbeitsgestaltung
- ☐ geringe soziale Unterstützung
- ☐ berufliche Unzufriedenheit
- ☐ Verlust des Arbeitsplatzes
- ☐ Kränkungsverhältnisse am Arbeitsplatz, chronischer Arbeitskonflikt (Mobbing)
- ☐ eigene negative Erwartung hinsichtlich der Rückkehr an den Arbeitsplatz

☐ Angst vor erneuter Schädigung am Arbeitsplatz

☐ keine Angabe

Weiter

Question 31 only appears if question 20 (screen 7) was answered with "Ja".

39% ausgefüllt

31. Erfragen Sie weitere Risikofaktoren für die Chronifizierung (z.B. geringe körperliche Aktivität)?

- ◯ Ja
- ◯ Nein

- ◯ keine Angabe

Weiter

B.Sc. Carolin Bahns B.Sc. Lisa Happe Hochschule für Gesundheit – 2017

Question 32 only appears if question 31 (screen 14) was answered with "Ja".

42% ausgefüllt

32. Welche weiteren Risikofaktoren erfragen Sie?

Mehrfachnennungen möglich

Iatrogene Faktoren
*Beeinflussung der Überzeugungen des/der Patienten*in sowie deren Umgang mit der Erkrankung durch die Überzeugungen und Einstellungen der behandelnden Ärzte*innen und Therapeuten*innen.*

- ☐ mangelhafte Respektierung der ganzheitlichen Krankheitsentstehung
- ☐ Überbewertung somatischer/radiologischer Befunde bei nicht-spezifischen Schmerzen
- ☐ lange, schwer begründbare Krankschreibung
- ☐ Förderung passiver Therapiekonzepte
- ☐ übertriebener Einsatz diagnostischer Maßnahmen

Sonstige Faktoren

- ☐ Rauchen
- ☐ Übergewicht
- ☐ geringe körperliche Kondition
- ☐ Alkohol

- ☐ keine Angabe

Weiter

Question 33 only appears if question 19 (screen 6) was answered with "Ja".

45% ausgefüllt

33. Führen Sie eine Untersuchung durch (bestehend aus z.B. Inspektion, Palpation und anderen Tests)?

- ◯ Ja
- ◯ Nein

- ◯ keine Angabe

Weiter

Question 34 only appears if question 33 (screen 16) was answered with "Ja".

hsg Hochschule für Gesundheit

48% ausgefüllt

34. Führen Sie eine Inspektion durch?

- ◯ Ja
- ◯ Nein

- ◯ keine Angabe

Weiter

Question 35 only appears if question 34 (screen 17) was answered with "Ja".

52% ausgefüllt

35. Welche Aspekte berücksichtigen Sie bei der Inspektion?

Mehrfachnennungen möglich

- ☐ Allgemeinzustand
- ☐ körperliche Beeinträchtigung
- ☐ Haltung
- ☐ Beckenstand
- ☐ Deformitäten
- ☐ Verletzungszeichen
- ☐ Haut

- ☐ keine Angabe

Weiter

Questions 36, 37, 38, 39 and 40 only appear if question 33 (screen 16) was answered with "Ja".

hsg Hochschule für Gesundheit

55% ausgefüllt

36. Führen Sie eine Palpation (der lokalen Muskulatur und der begleitend betroffenen Muskulatur auf Schmerzhaftigkeit und Verspannung) durch?

- ○ Ja
- ○ Nein

- ○ keine Angabe

37. Testen Sie lokalen Druck- oder Klopfschmerz der Processus spinosi (bei Verdacht auf Fraktur)?

- ○ Ja
- ○ Nein

- ○ keine Angabe

38. Führen Sie eine orientierende Beweglichkeitsprüfung (Ante-, Retro-, Lateralflexion der Lendenwirbelsäule) durch?

- ○ Ja
- ○ Nein

- ○ keine Angabe

39. Führen Sie eine Testung des Lasègue-Zeichens und ergänzend den Bragard-Test durch?

*Die Testung des Lasègue-Zeichens wird als eine Untersuchung auf Radikulopathie oder Nervendehnung verstanden: Durch Dehnung des Nervus ischiadicus bei passivem Anheben des gestreckten Beins des/der liegenden Patienten*in ausgelöster blitzartig einschießender Schmerz (Lasègue-Zeichen positiv) in Gesäß und Bein. Der Bragard-Test beinhaltet zusätzlich die passive Dorsalextension im Fußgelenk.*

- ○ Ja
- ○ Nein

- ○ keine Angabe

40. Führen Sie eine Untersuchung des Sakroiliakalen Gelenks (SIG) durch?

- ○ Ja
- ○ Nein

○ keine Angabe

Weiter

B.Sc. Carolin Bahns B.Sc. Lisa Happe Hochschule für Gesundheit – 2017

Question 41 only appears if question 40 (screen 19) was answered with "Ja".

58% ausgefüllt

41. Welche Untersuchungen des SIG führen Sie durch?

Mehrfachnennungen möglich

- ☐ lokale Schmerzpalpation
- ☐ Schmerzprovokation durch Kompression des Gelenkes

- ☐ keine Angabe

Weiter

Question 42 only appears if question 33 (screen 16) was answered with "Ja".

61% ausgefüllt

42. Führen Sie weitere Untersuchungsmaßnahmen durch?

- ◯ Ja, welche
- ◯ Nein

- ◯ keine Angabe

Weiter

Question 43 only appears if question 33 (screen 16) was answered with "Ja".

Hochschule für Gesundheit

64% ausgefüllt

43. Führen Sie bei Verdacht auf eine radikuläre Symptomatik ergänzend eine neurologische Untersuchung durch?

- ○ Ja
- ○ Nein

- ○ keine Angabe

Weiter

Question 44 only appears if question 43 (screen 22) was answered with "Ja".

67% ausgefüllt

44. Beurteilen Sie die Muskelkraft zum Nachweis von Paresen?

○ Ja
○ Nein

○ keine Angabe

Weiter

Question 45 only appears if question 44 (screen 23) was answered with "Ja".

Questions 46 and 47 only appear if question 43 (screen 22) was answered with "Ja".

hsg Hochschule für Gesundheit

70% ausgefüllt

45. Welche Untersuchungen der Muskelkraft zum Nachweis von Paresen führen Sie durch?

Mehrfachnennungen möglich

- ☐ Dorsalextension der Großzehe (L5)
- ☐ Dorsalflexion des Fußes im Sprunggelenk (L4 und L5)
- ☐ Plantarflexion des Fußes im Sprunggelenk (S1)
- ☐ Knieextension (L2-4)
- ☐ Hüftadduktion und -flexion gegen Widerstand (L1-4)

- ☐ keine Angabe

46. Führen Sie eine Überprüfung der Oberflächensensibilität durch Bestreichen der Haut an der unteren Extremität und im Gesäßbereich durch?

- ○ Ja
- ○ Nein

- ○ keine Angabe

47. Führen Sie eine Überprüfung der Muskeleigenreflexe durch?

- ○ Ja
- ○ Nein

- ○ keine Angabe

Weiter

Question 48 only appears if question 47 (screen 24) was answered with "Ja".

Hochschule für Gesundheit

73% ausgefüllt

48. Welche Muskeleigenreflexe überprüfen Sie?

Mehrfachnennungen möglich

- ☐ Achillessehnenreflex (S1)
- ☐ Patellasehnenreflex (L2-4)
- ☐ Babinski-Reflex (Abgrenzung von zentralen Läsionen)

- ☐ keine Angabe

Weiter

Hochschule für Gesundheit

76% ausgefüllt

III. Behandlung

49. Behandeln Sie Patienten*innen mit Kreuzschmerzen?

- ◯ Ja
- ◯ Nein

- ◯ keine Angabe

Weiter

Question 50 only appears if question 49 (screen 26) was answered with "Ja".

hsg Hochschule für Gesundheit

79% ausgefüllt

50. Welche der folgenden Behandlungsmethoden wenden Sie bei Patienten*innen mit akuten Kreuzschmerzen an, bzw. welche Behandlungsempfehlungen kommunizieren Sie mit dem/der Patienten*in?

Unter akuten Kreuzschmerzen werden neu aufgetretene Schmerzepisoden, die weniger als sechs Wochen anhalten, zusammengefasst.

	Nie	Manchmal	Oft	Immer	keine Angabe
Akupunktur	○	○	○	○	○
**Bewegung und Bewegungstherapie**					
➜ Krafttraining	○	○	○	○	○
➜ Ausdauertraining	○	○	○	○	○
Bettruhe	○	○	○	○	○
Entspannungsverfahren (progressive Muskelrelaxation)	○	○	○	○	○
Interferenzstromtherapie	○	○	○	○	○
Kinesio-Taping	○	○	○	○	○
Kurzwellendiathermie	○	○	○	○	○
Lasertherapie	○	○	○	○	○
Magnetfeldtherapie	○	○	○	○	○
**Manuelle Therapie**					
➜ Manipulation	○	○	○	○	○
➜ Mobilisation	○	○	○	○	○
Massage	○	○	○	○	○
**Medizinische Hilfsmittel**					
➜ Orthesen	○	○	○	○	○
➜ Schuheinlagen	○	○	○	○	○
Perkutane elektrische Nervenstimulation (PENS)	○	○	○	○	○
Rehasport und Funktionstrainig	○	○	○	○	○

Rückenschule (nach bio-psycho-sozialem Ansatz)	○	○	○	○	○
**Thermotherapie**					
➜ Kälte	○	○	○	○	○
➜ Wärme	○	○	○	○	○
Therapeutischer Ultraschall	○	○	○	○	○
Traktion mit Gerät	○	○	○	○	○
Transkutane elektrische Nervenstimulation (TENS)	○	○	○	○	○
Verhaltenstherapie (kognitiv)	○	○	○	○	○

Weiter

Question 51 only appears if question 49 (screen 26) was answered with "Ja".

Hochschule für Gesundheit

82% ausgefüllt

51. Welche der folgenden Behandlungsmethoden wenden Sie bei Patienten*innen mit chronischen Kreuzschmerzen an, bzw. welche Behandlungsempfehlungen kommunizieren Sie mit dem/der Patienten*in?

Unter chronischen Kreuzschmerzen werden Kreuzschmerzen, die länger als zwölf Wochen anhalten, zusammengefasst.

	Nie	Manchmal	Oft	Immer	keine Angabe
Akupunktur	○	○	○	○	○
**Bewegung und Bewegungstherapie**					
➜ Krafttraining	○	○	○	○	○
➜ Ausdauertraining	○	○	○	○	○
Bettruhe	○	○	○	○	○
Entspannungsverfahren (progressive Muskelrelaxation)	○	○	○	○	○
Interferenzstromtherapie	○	○	○	○	○
Kinesio-Taping	○	○	○	○	○
Kurzwellendiathermie	○	○	○	○	○
Lasertherapie	○	○	○	○	○
Magnetfeldtherapie	○	○	○	○	○
**Manuelle Therapie**					
➜ Manipulation	○	○	○	○	○
➜ Mobilisation	○	○	○	○	○
Massage	○	○	○	○	○
**Medizinische Hilfsmittel**					
➜ Orthesen	○	○	○	○	○
➜ Schuheinlagen	○	○	○	○	○
Perkutane elektrische Nervenstimulation (PENS)	○	○	○	○	○
Rehasport und Funktionstrainig	○	○	○	○	○

Rückenschule (nach bio-psycho-sozialem Ansatz)	○	○	○	○	○
**Thermotherapie**					
➜ Kälte	○	○	○	○	○
➜ Wärme	○	○	○	○	○
Therapeutischer Ultraschall	○	○	○	○	○
Traktion mit Gerät	○	○	○	○	○
Transkutane elektrische Nervenstimulation (TENS)	○	○	○	○	○
Verhaltenstherapie (kognitiv)	○	○	○	○	○

Weiter

85% ausgefüllt

IV. Wissenschaftlicher Bezug

52. Nutzen Sie im Rahmen Ihrer praktischen Berufsausübung als Physiotherapeut*in Empfehlungen aus Leitlinien?

Leitlinien sind definiert als systematisch entwickelte, wissenschaftlich begründete und praxisorientierte Entscheidungshilfen für die angemessene Vorgehensweise bei speziellen gesundheitlichen Problemen.

- ○ Ja
- ○ Nein

- ○ keine Angabe

Weiter

Question 53 only appears if question 52 (screen 29) was answered with "Ja".

88% ausgefüllt

53. Besprechen Sie Empfehlungen aus Leitlinien mit Ihren Patienten*innen?

○ Ja

○ Nein

○ keine Angabe

54. Ist Ihnen die Nationale Versorgungsleitlinie (NVL) Nicht-spezifischer Kreuzschmerz, 2. Auflage (Link zur NVL: http://www.leitlinien.de/nvl/kreuzschmerz), bekannt, bzw. haben Sie sich mit dieser auseinandergesetzt?

Der Link öffnet sich in einem neuen Fenster.

○ Ja

○ Nein

○ keine Angabe

Weiter

Questions 53 and 55 only appear if question 52 (screen 29) was answered with "No".

91% ausgefüllt

53. Aus welchen Gründen?

Mehrfachnennungen möglich

- ☐ Empfehlungen aus Leitlinien sind nicht hilfreich, um die Patientenversorgung zu verbessern
- ☐ Empfehlungen aus Leitlinien widerprechen meiner klinischen Expertise
- ☐ der Einsatz von Leitlinien wird an meiner Arbeitsstelle nicht unterstützt
- ☐ Empfehlungen aus Leitlinien werden nicht der Individualität der Patienten*innen gerecht
- ☐ Empfehlungen aus Leitlinien behindern mich in meiner Entscheidungsfindung
- ☐ Ich habe keine Zeit, um Leitlinien zu lesen
- ☐ Ich weiß nicht, wo ich Leitlinien finden kann
- ☐ Sonstiges:

- ☐ keine Angabe

54. Ist Ihnen die Nationale Versorgungsleitlinie (NVL) Nicht-spezifischer Kreuzschmerz, 2. Auflage (Link zur NVL: http://www.leitlinien.de/nvl/kreuzschmerz), bekannt, bzw. haben Sie sich mit dieser auseinandergesetzt?

Der Link öffnet sich in einem neuen Fenster.

- ○ Ja
- ○ Nein

- ○ keine Angabe

55. Haben Sie generelles Interesse, Empfehlungen aus Leitlinien in Ihre tägliche Arbeit mit Patienten*innen einzubeziehen?

- ○ Ja
- ○ Nein

- ○ keine Angabe

Weiter

Hochschule für Gesundheit

94% ausgefüllt

56. Wie sind Sie auf diese Befragung aufmerksam geworden?

- ○ Arbeitgeber
- ○ Freunde und Bekannte
- ○ Kollegen
- ○ Aufruf im Verbandsnetzwerk
- ○ Aufruf über soziale Medien
- ○ Aufruf in Zeitschrift
- ○ Information der Hochschule für Gesundheit Bochum
- ○ Sonstiges: ______

57. An dieser Stelle ist Platz für Kommentare, Anmerkungen oder Kritik zur Studie:

Weiter

94% ausgefüllt

☐ Wenn Sie über die **Ergebnisse der Studie** informiert werden möchten, geben Sie hier bitte Ihre E-Mail Adresse an.
Ihre E-Mail Adresse wird sicher verwahrt und lässt keinen Rückschluss auf Ihre genannten Informationen zu.

Weiter

Vielen Dank für Ihre Teilnahme!

Wir möchten uns ganz herzlich für Ihre Mithilfe bedanken.

Ihre Antworten wurden gespeichert, Sie können das Browser-Fenster nun schließen.
