# Supplementary material for: Physical therapy for patients with low back pain in Germany: a survey of current practice
Source: BMC Musculoskelet Disord. 2021 Jun 19;22:563. doi: 10.1186/s12891-021-04422-2 (PMC8214788; doi:10.1186/s12891-021-04422-2)
Supplement: Supplementary file 2 — Additional file 2. Translated English language version of the questionnaire [file 12891_2021_4422_MOESM2_ESM.pdf]

Please note: This English language version is a direct translation of the original questionnaire without any form of cultural adaptation.

Dear Participants,

Thank you very much for your interest in our online survey on "Physical therapy for patients with low back pain in Germany."

What is the study about?

We would like to ask you a few questions about the following four subject areas:

1. General demographic and occupational information
2. Contents and scope of the physical therapeutic diagnostic process of patients with acute and chronic low back pain
3. Contents and recommendations for the treatment of patients with acute and chronic low back pain
4. Knowledge and application of clinical practice guidelines in the physical therapy practice

What is the aim of the study?

The aim of the study is to evaluate the current physical therapy management of patients with low back pain in Germany with regard to the recommendations of the National Disease Management Guideline (NVL) "Non-specific low back pain", 2nd edition.

How much time does it take to participate?

Participation in the online survey takes about **15 minutes**.

What happens with my data?

The gathered data will be kept under lock and key and be published only in anonymised form for scientific purposes.

What are the risks?

The survey is not associated with any risks.

Your participation in this study is voluntary. If you decide to take part, you are still free to withdraw yourself and your data from the study without giving a reason. You are not obligated to answer all questions and you can stop the survey at any time.

Data Privacy Statement

§ I grant my consent to the recording of the data gathered in the course of the study on electronic data carriers and their storing in pseudonymised form* for scientific analysis at the data gathering and analysis centre**.

§ Furthermore, I also consent to an authorised agent of the ethics committee, who is obligated for confidentiality, accessing my personal data, which is available at the data gathering and analysis centre**, insofar as this is necessary for the validation of the study. For these measures, I release the data gathering and analysis centre from its confidentiality obligation.

§ I am informed that I can withdraw from the study at any time without this causing any negative effects for me.

* Pseudonymisation means replacing the name and other identifying characteristics by a code for the purpose of preventing the identification of the data subject or making this significantly more difficult (Sec. 3 (6a) BDSG [German Federal Data Protection Act]).

** Prof. Dr Christian Kopkow, University of Applied Sciences, Department of Applied Health Sciences, Gesundheitscampus 6-8, 44801 Bochum

I have understood the participant information on the study "Physical therapy for patients with low back pain in Germany" and I consent to participate in the study and to the pseudonymised recording and analysis of my data, the transfer the data to others and the examination of my personal data in the form described above.

Click **"Continue"** to participate in the study.

Note

Please note that the survey must be completed in one go to prevent the loss of data (**it is not possible to save the results to temporary storage**). We also want to inform you that as soon as you click on "Continue", you **cannot** return to the previous page of the survey.

Therefore, please take enough time to read the following questions and answer them to the best of your knowledge and belief.

Thank you very much for participating in this important study!

I. General demographic and occupational information

1. How old are you?

___ years ◻ No answer

2. What is your gender?

○ Male ○ Female ○ Other ○ No answer

3. In what type of practice do you work?

(Multiple answers are allowed)

◻ Private practice

◻ Hospital

◻ Rehabilitation clinic

◻ Other: ______

◻ No answer

4. In what federal state of Germany do you work?

[please select]

5. How many inhabitants live in the town (or municipality) of your workplace?

If you have more than one place of work, please answer this question with regard to the workplace where you work most of the time.

○ <5,000 inhabitants (rural area)

○ 5,000 - 20,000 inhabitants (small town)

○ 20,000 - 100,000 inhabitants (mid-sized town)

○ > 100,000 inhabitants (large town)

○ No answer

6. Are you:

If you have more than one place of work as physical therapist, please answer this question with regard to your primary employment.

○ Employee/worker ○ Self-employed ○ Other ○ No answer

(Note: Questions 7 and 8 only appear if question 6 was answered with "Employee/worker".)

7. What is your current employment contract situation?

○ Permanent employment ○ Fixed-term employment ○ No answer

8. How many colleagues work with you in a team?

(only physical therapists)

_____ colleagues □ No answer

(Note: Question 9 only appears if question 6 was answered with "Self-employed".)

9. How many employees work in your team?

_____ employees □ No answer

10. How many hours per week do you work as physical therapist?

_____ hours/week □ No answer

11. Where did you complete your vocational training?

○ Vocational school

○ University

○ Other (e.g. university abroad, occupational degree programme, ...)

○ No answer

12. Which of the following degrees have you attained in the field of healthcare to date?

(Multiple answers are allowed)

□ Diploma (vocational school)

□ Diploma (university)

□ Bachelor

□ Master

□ Doctorate

□ No answer

13. How many years have you worked as a physical therapist?

_____ year(s) □ No answer

14. How many new patients with low back pain do you usually see during one week?

_____ patients per week □ No answer

15. Have you completed one or more of the following advanced trainings?

(Multiple answers are allowed)

□ Manual therapy (MT)

□ Orthopaedic manual therapy (OMT)

□ Healing practitioner

□ Osteopathy

□ Back school

□ Pain management physiotherapy

□ Other: ____ *(Please indicate only advanced trainings with relevance for back pain.)*

□ None

□ No answer

16. Are you engaged in direct communication with other healthcare professionals at your organisation (physicians, psychologists, occupational therapists, ...)?

o Yes o No o No answer

(Note: Questions 17 only appears if question 16 was answered with "Yes".)

17. With which professional groups do you communicate at your workplace?

(Multiple answers are allowed)

□ Physicians

□ Psychotherapists

□ Nursing staff

□ Occupational therapists

□ Other: ____

□ No answer

18. How much time do you have available in the course of a normal workday for each of the following activities?

Initial physical therapeutic diagnostic: ____ min □ No answer

Treatment: ____min □ No answer

II. Physical therapeutic diagnostic process

19. Do you differentiate between acute and chronic low back pain?

Acute low back pain summarises newly occurring pain episodes, which persist for less than six weeks. Chronic low back pain refers to back pain, which persists for more than twelve weeks.

○ Yes

○ No

○ No answer

20. Do you conduct an initial physical therapeutic diagnostic process?

○ Yes

○ No

○ No answer

(Note: Question 21 only appears if question 20 was answered with "Yes".)

21. Do you evaluate medical history?

○ Yes

○ No

○ No answer

22. Do you ask about pain characteristics (e.g. localisation, beginning, course, ...)?

○ Yes

○ No

○ No answer

(Note: Question 23 only appears if question 22 was answered with "Yes".)

23. Which characteristics do you ask about?

(Multiple answers are allowed)

□ Localisation and radiation

□ Beginning

□ Provoking, aggravating or relieving factors

□ Progression over time (of the day)

□ Limitations in daily activities

□ Previous episodes

□ No answer

(Note: Question 24 only appears if question 21 was answered with “Yes”.)

24. Do you ask about extra-spinal causes?

Extra-spinal causes are defined as pain in the area of the lumbar region that is triggered by neighbouring organs, which are not directly part of the osseous, muscular or spinal ligament structures (e.g. caused by an aortic aneurysm).

o Yes

o No

o No answer

(Note: Question 25 only appears if question 24 was answered with “Yes”.)

25. Which extra-spinal causes do you ask about?

(Multiple answers are allowed)

□ Abdominal and visceral processes (e.g. pancreatitis)

□ Vascular injuries (e.g. aortic aneurysms)

□ Gynaecological causes (e.g. endometriosis)

□ Urological causes (e.g. urolithiasis)

□ Neurological diseases (e.g. polyneuropathy)

□ Psychosomatic and psychiatric disorders

□ No answer

(Note: Questions 26, 27 and 28 only appear if question 21 was answered with “Yes”.)

26. Do you ask about “red flags”?

“Red flags” are defined as signs and suspicions on a specific aetiology of low back pain, which require clarification and therapy by a physician soon and, if necessary, on an emergency basis.

o Yes

o No

o No answer

27. Do you ask about “yellow flags”?

“Yellow flags” are defined as psychosocial factors, which increase the risk of the low back pain becoming chronic and which play a decisive role in the course of the condition.

o Yes

o No

○ No answer

28. Do you ask about “blue” and “black flags”?

“Blue” and “black flags” are defined as factors specific to the workplace, which influence the development of chronic low back pain.

○ Yes

○ No

○ No answer

(Note: Questions 29, 30 and 31 only appear if questions 26, 27 and 28 were answered with “Yes”.)

29. When you ask for “red flags”, which of the following pathologies do you consider?

(Multiple answers are allowed)

□ Fracture/osteoporosis

□ Infection

□ Radiculopathies/neuropathies

□ Tumour/metastases

□ Axial spondyloarthritis

□ No answer

30. Which “yellow flags” do you ask about?

(Multiple answers are allowed)

□ Depressiveness/distress (negative stress)

□ Pain-related cognitions (e.g. fear-avoidance beliefs, catastrophizing)

□ Passive pain behaviour (e.g. strong tendency to spare oneself and avoidance behaviour)

□ Overactive pain behaviour (task persistence)

□ Pain-related cognitions (thought suppression)

□ Tendency toward somatisation (occurrence of physical ailments, which have no organic cause but which have psychological causes)

□ No answer

31. Which “blue” and “black flags” do you ask about?

Multiple answers are allowed

□ Predominantly heavy physical labour

□ Predominantly monotonous body posture

□ Predominantly exposure to vibrations

□ unskilled work

□ Little influence on the arrangement of work

□ Little social support

□ Work dissatisfaction

□ Loss of employment

□ Offensive work relationships, harassment

□ Own negative expectation as relates to returning to the job

□ Fear of repeated degradation on the job

□ No answer

(Note: Question 32 only appears if question 21 was answered with "Yes".)

32. Do you ask about further risk factors for chronification (e.g. poor physical fitness)?

○ Yes

○ No

○ No answer

(Note: Question 33 only appears if question 32 was answered with "Yes".)

33. Which further risk factors do you ask about?

(Multiple answers are allowed)

Iatrogenic factors

These are defined to mean the manipulation of patients' beliefs and their handling of the illness based on the beliefs and attitudes of the treating physicians and therapists.

□ Insufficient respect for a multifactorial causality

□ Overestimating somatic/radiologic findings in the case of non-specific pain

□ prolonged sick leave that is hard to justify

□ Promoting passive therapy concepts

□ Excessive use of diagnostic procedures

Other factors

□ Smoking

□ Obesity

□ Poor physical fitness

□ Alcohol

□ No answer

(Note: Question 34 only appears if question 20 was answered with "Yes".)

34. Do you conduct a physical examination (consisting, e.g., of an inspection, palpation and other tests)?

○ Yes

○ No

○ No answer

(Note: Question 35 only appears if question 34 was answered with "Yes".)

35. Do you conduct an inspection?

○ Yes

○ No

○ No answer

(Note: Question 36 only appears if question 35 was answered with "Yes".)

36. Which aspects do you consider in the inspection?

(Multiple answers are allowed)

□ General condition

□ Physical impairments

□ Posture

□ Pelvic stand

□ Deformities

□ Signs of injury

□ Skin

□ No answer

(Note: Questions 37, 38, 39, 40 and 41 only appears if question 34 was answered with "Yes".)

37. Do you conduct a palpation (of local musculature and the secondarily afflicted musculature for pain sensation and tension)?

○ Yes

○ No

o No answer

38. Do you test local tenderness of the spinal process on pressure or percussion (in case of a suspected fracture)?

o Yes

o No

o No answer

39. Do you carry out an orienting movement testing (anteflexion, retroflexion, lateral flexion of the lumbar spine)?

o Yes

o No

o No answer

40. Do you test the straight leg raise and carry out the Bragard test in addition?

Testing the straight leg raise is understood to mean an exam for radiculopathy or nerve flexion: flashing pain in the buttocks and leg triggered suddenly by flexing the sciatic nerve when raising the straight leg passively (positive indication of the straight leg raise). The Bragard Test includes additionally the passive dorsal extension in the ankle of the foot.

o Yes

o No

o No answer

41. Do you examine the sacroiliac joint?

o Yes

o No

o No answer

(Note: Question 42 only appears if question 41 was answered with "Yes".)

42. Which exams of the sacroiliac joint do you conduct?

(Multiple answers are allowed)

□ Local pain palpation

□ Pain provocation by compression of the joint

□ No answer

(Note: Question 43 only appears if question 34 was answered with "Yes".)

43. Do you conduct further exam measures?

○ Yes, which ones _____

○ No

○ No answer

(Note: Question 44 only appears if question 34 was answered with "Yes".)

44. In case of suspected radicular symptoms, do you additionally conduct a neurological examination?

○ Yes

○ No

○ No answer

(Note: Question 45 only appears if question 44 was answered with "Yes".)

45. Do you evaluate the muscle strength to detect paresis?

○ Yes

○ No

○ No answer

(Note: Question 46 only appears if question 45 was answered with "Yes".)

46. Which tests of the muscle strength do you carry out to detect paresis?

(Multiple answers are allowed)

□ Dorsal extension of the big toe (L5)

□ Dorsal flexion of the foot in the ankle joint (L4 and L5)

□ Plantar flexion of the foot in the ankle joint (S1)

□ Knee extension (L2-4)

□ Hip adduction and flexion against resistance (L1-4)

□ No answer

(Note: Questions 47 and 48 only appear if question 44 was answered with "Yes".)

47. Do you check for sensory disorder by gently brushing the skin on the lower extremities and the buttocks region?

○ Yes

○ No

○ No answer

48. Do you test the muscle reflexes?

○ Yes

○ No

○ No answer

(Note: Question 49 only appears if question 48 was answered with "Yes".)

49. Which muscle reflexes do you test?

(Multiple answers are allowed)

□ Hamstring reflex (S1)

□ Patellar reflex (L2-4)

□ Babinski reflex (differentiation from central lesions)

□ No answer

III. Treatment

50. Do you treat patients with low back pain?

○ Yes

○ No

○ No answer

(Note: Question 51 only appears if question 50 was answered with "Yes".)

51. Which of the following treatment modalities do you use for patients with acute low back pain or which treatment recommendations do you communicate to the patient?

Acute low back pain summarises newly occurring pain episodes, which persist for less than six weeks.

	Never	Sometimes	Often	Always	No answer
Acupuncture	○	○	○	○	○
**Exercise and exercise therapy**<br>➔ Strength training<br>➔ Endurance training	○	○	○	○	○
Bed rest	○	○	○	○	○
Relaxation techniques (progressive muscle relaxation)	○	○	○	○	○
Interference therapy	○	○	○	○	○
Kinesio taping	○	○	○	○	○
Short-wave diathermy	○	○	○	○	○
Laser therapy	○	○	○	○	○
Magnetic field therapy	○	○	○	○	○
**Manual therapy**<br>➔ Spinal manipulation<br>➔ Spinal mobilisation	○	○	○	○	○
Massage	○	○	○	○	○
**Medical aids**<br>➔ Orthoses<br>➔ Orthopedic insoles	○	○	○	○	○
Percutaneous electrical nerve stimulation (PENS)	○	○	○	○	○
Rehabilitation sport and functional training	○	○	○	○	○
Back school (according to a bio-physical-social approach)	○	○	○	○	○
**Thermotherapy**<br>➔ Cold therapy<br>➔ Heat therapy	○	○	○	○	○
Ultrasound therapy	○	○	○	○	○
Instrumental traction	○	○	○	○	○
Transcutaneous electrical nerve stimulation (TENS)	○	○	○	○	○
Behavioural therapy	○	○	○	○	○

(Note: Question 52 only appears if question 50 was answered with "Yes".)

52. Which of the following treatment methods do you use for patients with chronic low back pain or which treatment recommendations do you communicate to the patient?

Chronic low back pain summarises back pain, which persists for more than twelve weeks.

	Never	Sometimes	Often	Always	No answer
Acupuncture	o	o	o	o	o
**Exercise and exercise therapy**<br>➜ Strength training<br>➜ Endurance training	o	o	o	o	o
Bed rest	o	o	o	o	o
Relaxation techniques (progressive muscle relaxation)	o	o	o	o	o
Interference therapy	o	o	o	o	o
Kinesio taping	o	o	o	o	o
Short-wave diathermy	o	o	o	o	o
Laser therapy	o	o	o	o	o
Magnetic field therapy	o	o	o	o	o
**Manual therapy**<br>➜ Spinal manipulation<br>➜ Spinal mobilisation	o	o	o	o	o
Massage	o	o	o	o	o
**Medical aids**<br>➜ Orthoses<br>➜ Orthopedic insoles	o	o	o	o	o
Percutaneous electrical nerve stimulation (PENS)	o	o	o	o	o
Rehabilitation sport and functional training	o	o	o	o	o
Back school (according to a bio-physical-social approach)	o	o	o	o	o
**Thermotherapy**<br>➜ Cold therapy<br>➜ Heat therapy	o	o	o	o	o
Ultrasound therapy	o	o	o	o	o
Instrumental traction	o	o	o	o	o
Transcutaneous electrical nerve stimulation (TENS)	o	o	o	o	o
Behavioural therapy	o	o	o	o	o

IV. Guidelines

53. Do you use recommendations from guidelines in your clinical practice as a physical therapist?

Guidelines are defined as systematically developed, scientifically reasoned and practice-relevant aids for making decisions on the appropriate approach to specific health conditions.

o Yes

o No

o No answer

(Note: Question 54 only appears if question 53 was answered with "Yes".)

54. Do you discuss the recommendations from guidelines with your patients?

o Yes

o No

o No answer

55. Do you know the National Versorgungsleitlinie [National Care Guideline] ("NVL") Non-specific pack pain, 2nd edition (link to the NVL: http://www.leitlinien.de/nvl/kreuzschmerz) and have dealt with its recommendations, respectively?

The link opens a new window.

o Yes

o No

o No answer

(Note: Questions 56 and 57 only appear if question 53 was answered with "No".)

56. For what reasons?

(Multiple answers are allowed)

□ Recommendations from guidelines are not helpful to improve the patient care

□ Recommendations from guidelines contradict my own clinical expertise

□ Using guidelines is not supported at my workplace

□ Recommendations from guidelines are not suitable given the individuality of the patients

□ Recommendations from guidelines are hindrances to my decision-making

□ I have no time to read guidelines

□ I do not know where I can find guidelines

□ Other: _____

□ No answer

57. Do you have a general interest in considering recommendations from guidelines in your daily work with patients?

○ Yes

○ No

○ No answer

58. How have you first heard of this survey?

○ Employer

○ Friends and acquaintances

○ Colleagues

○ Call for participants in the association network

○ Call for participants in social media

○ Call for participants in a magazine

○ Information from the University of Applied Sciences in Bochum

○ Other: ____

59. You have the opportunity here to add remarks, comments or criticism about the study:

____

Thank you very much for your participation!

We would like to thank you very much for your help.

Your answers have been saved and you can now close the browser window.
